# Descriptions of the mature larva and pupa of the Scaly strawberry weevil, *Sciaphilus asperatus* (Bonsdorff, 1785) (Coleoptera, Curculionidae, Entiminae) and observations of its biology

**DOI:** 10.3897/zookeys.873.35922

**Published:** 2019-08-29

**Authors:** Rafał Gosik, Peter Sprick, Tetiana Tiahunova

**Affiliations:** 1 Department of Zoology, Maria Curie–Skłodowska University, Akademicka 19, 20–033 Lublin, Poland Skłodowska University Lublin Poland; 2 Curculio–Institute e.V. (CURCI), Weckenstraße 15, 30451 Hannover, Germany Curculio - Institute e.V. (CURCI) Hannover Germany; 3 Olzhycha 8/51, 04086 Kiev, Ukraine Unaffiliated Kiev Ukraine

**Keywords:** Biology, chaetotaxy, determination, host plants, larval instar, life history, morphology, plant protection, weevils

## Abstract

The mature larva of *Sciaphilus asperatus* is redescribed and illustrated, and the pupa is described for the first time. Supplements to the identification keys for larvae and pupae of selected Palaearctic Entiminae genera and species are given. Data on the life history, especially oviposition capacity and voltinism, of *S. asperatus* are provided and discussed, and the number of the six larval instars was confirmed. The economic importance of *S. asperatus* is briefly highlighted.

## Introduction

The weevil genus *Sciaphilus* Schönherr, 1823 contains five valid species: *S. humeralis* Desbrochers des Loges, 1902 occurs in North Africa, *S. helenae* Schilsky, 1912 in the Middle East, *S. costulatus* Kiesenwetter, 1852 and *S. ebeninus* Chevrolat, 1873 are known from isolated localities in Europe, while *S. asperatus* (Bonsdorff, 1785), the species treated in this paper, is widespread in the Western Palaearctic, Central Asia (Kazakhstan), and western Siberia (Tomsk region) (Morris 1997; Legalov 2010; Borovec 2013; Alonso-Zarazaga et al. 2017). It was recently accidentally introduced to North America (Bright and Bouchard 2008).

**Figures 1–3.**
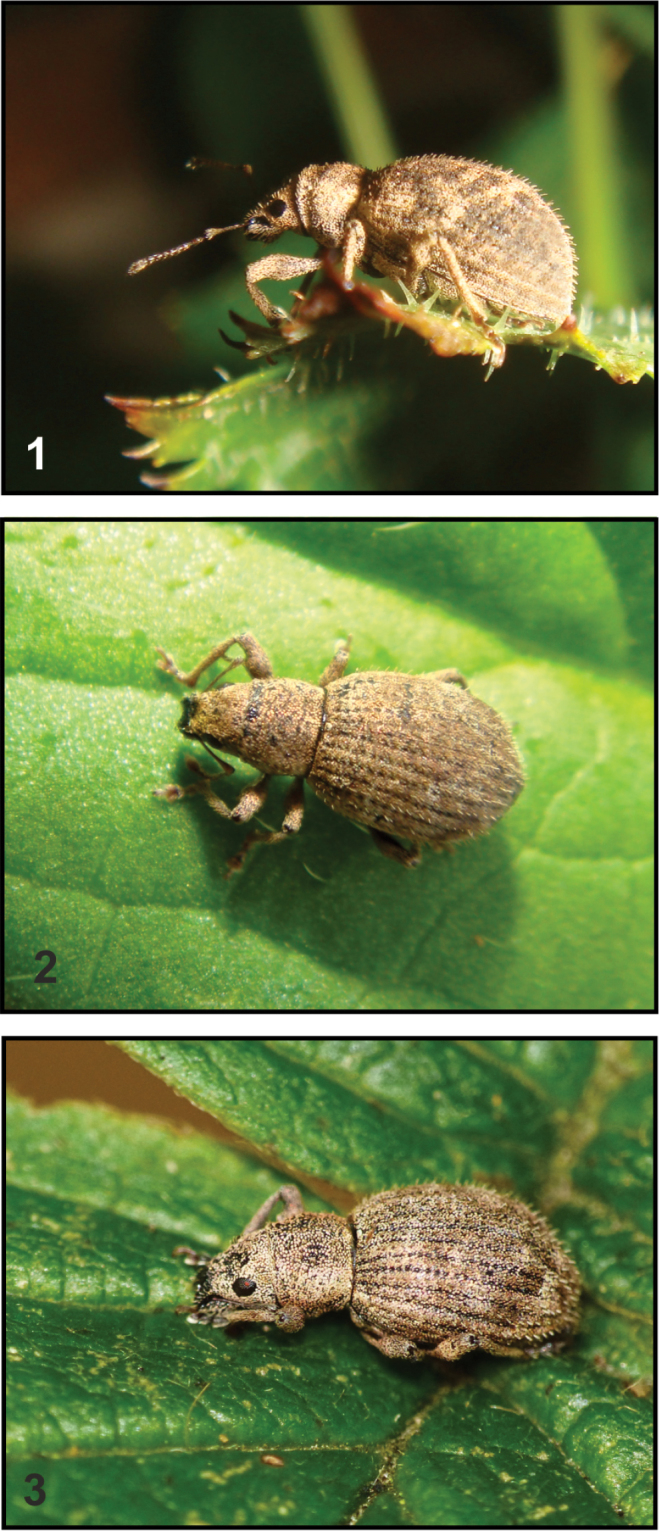
*Sciaphilus asperatus* adult, field photographs (photograph P. Sprick).

**Figure 4.**
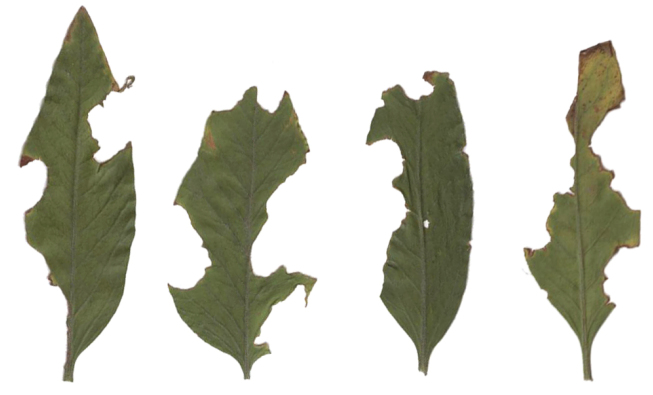
Leaves of *Weigela florida* with traces of feeding by adults of *Sciaphilus asperatus* (photograph R. Gosik).

Species of *Sciaphilus* form a rather uniform group characterized in the adult stage by: (1) small body size (< 6 mm); (2) short rostrum with acute carina close to apex; (3) flat eyes; (4) long, slender antennae; (5) rounded elytra densely covered with oblong, erect and spherical adherent scales, the latter forming a more or less contrasting pattern; (6) femora with a conspicuous tooth (Hoffmann 1950; Smreczyński 1966; Freude et al. 1981). *Sciaphilus asperatus* (Figs 1–3) is a wingless, parthenogenetic, triploid species (Suomalainen 1969; Morris 1997). The adult is a polyphagous feeder on leaves of many herbs, shrubs and trees, mainly in the herb or even in the lower shrub layer, producing more or less characteristic notches on the leaf edge (Fig. 4). In the larval stage it feeds on the roots of plants like strawberry (*Fragaria* L.), cinquefoil (*Potentilla* L.), raspberry, blackberry (both *Rubus* L.), hawthorn (*Crataegus* L., all Rosaceae), and primrose (*Primula* L., Primulaceae). In the Berggarten area of Hannover-Herrenhausen it was regularly found in beds of *Astilbe* Buch.-Ham. ex D. Don, *Tiarella* L. (both Saxifragaceae), *Epimedium* L. (Berberidaceae) and small *Rhododendron* L. species (Ericaceae) (Sprick and Stüben 2012). In Lublin adults of *S. asperatus* were observed feeding also on *Weigela florida* (Bunge) DC. (Caprifoliaceae). In the laboratory, adults readily fed on a great number of plant species from more than 15 families (Willis 1964; Dieckmann 1980; Burakowski et al. 1993; Sprick and Stüben 2012), many of which may also be host plants. It is a eurytopic species reported from a large variety of biotopes, preferring rather moist and shady places. It occurs mostly in forests, bushes, fallow grassland and on river banks, but also in cultivations, like tree nurseries, parks and gardens (Koch 1992; Burakowski et al. 1993; Morris 1997; Gosik 2007; Sprick and Stüben 2012).

Biology and life-cycle of *Sciaphilus asperatus* have been described by Willis (1964), Krause (1978), Dieckmann (1980), and Burakowski et al. (1993). Adults are observed on host plants from mid-April to the beginning of October (Dieckmann 1980). The oviposition period in the field extends from late April to the end of July. Eggs are laid in batches between adjoining surfaces which was stated by Marvaldi (1999a) to be a common oviposition type in many Entiminae genera. Willis (1964) reported egg masses of 6 to 157 eggs, while Dieckmann (1980) recorded ca. 80 eggs (several observations). In the laboratory, we observed masses of 21 and 95 eggs (Fig. 23), deposited in two or three rows and glued with a secretion between layers of filter paper, or between the paper or a leaf placed in the box as food supply and the substrate. In the field, eggs are glued between overlapping leaves, to leaf folds, leaf petioles and stems, usually close to the ground (Willis 1964). Dieckmann (1980) and Willis (1964) reported that per year a single specimen can produce up to 880 or to 1000 eggs, respectively.

The larva develops in spring and summer, and this species usually overwinters in the adult stage (Krause 1978). According to Willis (1964), a small number of newly emerged weevils lay eggs – after a rather long pre-oviposition period of 24–31 days (only 12 days in spring) – also between mid-August and the beginning of September, which approximates the life-cycle of *S. asperatus* to that of many other soil-dwelling weevils by the presence of overwintering adults and larvae (see Gosik et al. 2016, Gosik et al. 2019). *S. asperatus* pupates between June and August; pupation in the field lasts between 14 and 21 days (Willis 1964).

The economic importance of *Sciaphilus asperatus* is usually low, compared to that of several *Otiorhynchus* species. Willis (1964) reported only two cases of severe damage in commercial strawberry cultivations in Northern Ireland and concluded: “From the limited data available it appears probable that severe damage to strawberry plants by larvae of *S. asperatus* occurs infrequently in Northern Ireland and tends to be associated with areas of light, well-drained soil.” Alford (1999) also restricted the potential economic importance to strawberries: “Weevils from related genera [other than *Otiorhynchus*] (e.g. *Exomias* Bedel, 1883 and *Sciaphilus*) are also of pest status, e.g. on strawberry.” Sprick and Stüben (2012), who studied the soil-dwelling weevil fauna of many tree nurseries, garden centres and parks in Germany, also ranked this species in the category of minor economic importance: “Species that usually rarely cause damage”. In a study from North America, *S. asperatus* comprised nearly 10% of the total larvae in forest soils (ten sites) from the Great Lakes region in Michigan and Wisconsin (Pinski et al. 2005). *S. asperatus* sometimes forms rather large populations, which is possible, given its parthenogenesis and oviposition capacity, but Morris (1997) stated that this species rarely occurs in large numbers in Great Britain. In a small number of cultivations, mainly of Rosaceae herbs and preferably strawberries, it achieved large numbers and therefore pest status, too.

The morphology of immatures of *Sciaphilus asperatus* is still incompletely known. A piece of information on this topic is given by Emden (1950, 1952): his paper contains descriptions of spiracles and the body shape of first larval instars, as well as diagnostic characters at genus and species level. Moreover, differences between first instar larva and the mature larva are provided. But only diagrams of labrum and epipharynx of the first instar larva are presented. On the other hand, the pupa remained still unknown. On the basis of head measurements, Willis (1964) reported the presence of six larval instars in *S. asperatus*.

## Materials and methods

### Specimens examined

Eleven mature larvae; eight pupae, 11.07.2013, Hannover-Herrenhausen, Berggarten, botanical garden, collected from a bed of *Waldsteinia geoides* Willd. (Rosaceae).

All the larvae and pupae were collected in the field at a site where the life cycle had previously been studied using pitfall traps (see Sprick and Stüben 2012). The field work in the consecutive season was concentrated on obtaining materials for morphological study. The mature larva and pupa were described, whereas the first instar larva was used only for measurement purposes in order to ascertain the number of developmental stages (see for example Sprick and Gosik 2014 or Gosik et al. 2019). Immature stages were preserved in 75% ethanol and used for measurements and morphological descriptions.

Slide preparation basically followed May (1994). The larvae selected for study under the microscope were cleared in 10% potassium hydroxide (KOH), then rinsed in distilled water and dissected. After clearing, head, mouthparts and body (thoracic and abdominal segments) were separated and mounted on permanent microscope slides in Faure-Berlese fluid (50 g Gum Arabic and 45 g chloral hydrate dissolved in 80 g of distilled water and 60 cm^3^ of glycerol) (Hille Ris Lambers 1950). The specimens and slides are deposited in the collections of the Department of Zoology, Maria Curie-Skłodowska University (Lublin, Poland).

The study was conducted using a light compound microscope (Ampliwal) with calibrated oculars and a drawing tube (MNR–1). Drawings and outlines were processed by computer software (Corel Photo-Paint X6, Corel Draw X6). The photographs were taken with an Olympus BX63 microscope and processed by Olympus cellSens Dimension software. We follow the chaetotaxy nomenclature proposed by Anderson (1947), Scherf (1964), May (1994), Marvaldi (1997, 1998, 1999b, 2003) and Skuhrovec et al. (2015), with the antennae terminology following Zacharuk (1985).

### Morphological abbreviations

**Larva**

**Abd. 1–10** abdominal segments 1–10,

**at** antenna,

**clss** clypeal sensorium,

**st** stemmata,

**Se** sensorium,

**sa** sensillum ampullaceum,

**sb** sensillum basiconicum,

**sc** sensilla cluster,

**lr** labral rods,

***als*** anterolateral seta,

***ams*** anteromedial seta,

***as*** alar seta,

***cls*** clypeal seta,

***des*** dorsal seta,

***dms*** dorsal malar seta,

***ds*** dorsal seta,

***eps*** epipleural seta,

***eus*** eusternal seta,

***fs*** frontal seta,

***les*** lateral epicranial seta,

***ligs*** ligular seta,

***lrs*** labral seta,

***ls*** lateral seta,

***lsts*** laterosternal seta,

***mbs*** malar basiventral seta,

***mds*** mandibular seta,

***mes*** median seta,

***mps*** maxillary palp seta,

***pda*** pedal seta,

***pds*** postdorsal seta,

***pes*** postepicranial seta,

***pfs*** palpiferal seta,

***pms*** postlabial seta,

***prms*** prelabial seta,

***prns*** pronotal seta,

***prs*** prodorsal seta,

***ps*** pleural seta,

***ss*** spiracular seta,

***stps*** stipal seta,

***sts*** sternal seta,

***ts*** terminal seta,

**Th. 1–3** thoracic segments 1–3,

***vms*** ventral malar seta.

**Pupa**

***as*** apical seta,

***d*** dorsal seta,

***ds*** discal seta,

***es*** epistomal seta,

***fes*** femoral seta,

***os*** orbital seta,

***pas*** postantennal seta,

***rs*** rostral seta,

***sls*** superlateral seta,

***sos*** superorbital seta,

***ss*** spiracular seta,

**ur** urogomphi,

***v*** ventral seta,

***vs*** vertical seta.

## Results

### Description of the mature larva

All data in [mm], (^n^: number of exemplars).

First instar larvae: Head width 0.224^1^, 0.230^1^.

Mature larvae: Head width 1.05^1^, 1.10^5^, 1.15^3^, 1.17^2^; body length: 3.50^1^, 4.00^2^; 4.50^1^, 5.00^2^, 5.50^2^, 6.00^3^; body width: 1.50^5^, 1.75^2^, 2.00^4^.

**Figures 5–9.**
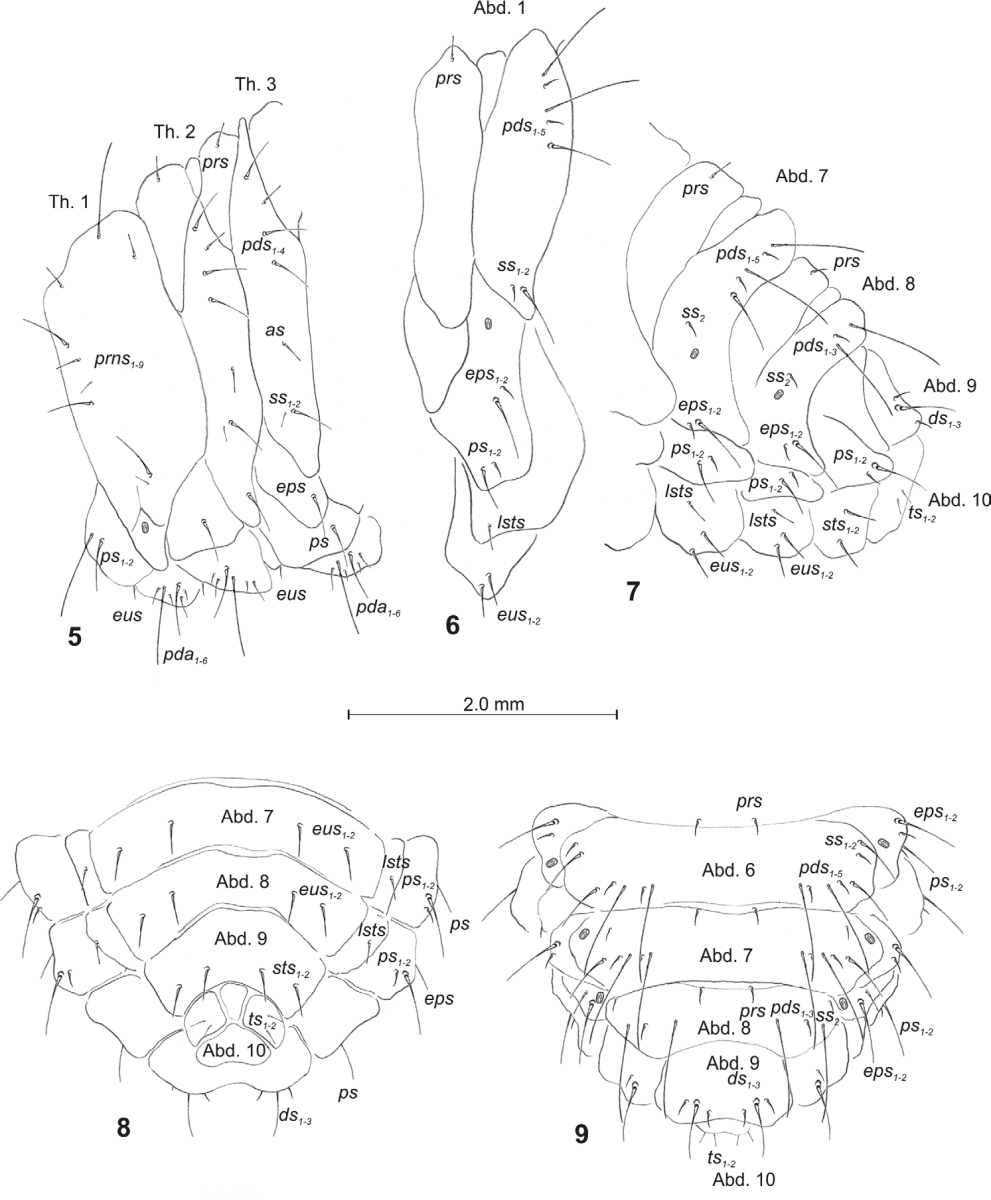
*Sciaphilus asperatus* mature larva, habitus and chaetotaxy. **5** Thoracic segments, lateral view **6** First abdominal segment, lateral view **7** Abdominal segments 7–10, lateral view **8** Abdominal segments 7–10, ventral view **9** Abdominal segments 6–10, dorsal view. Abbreviations: Th. 1–3 – thoracic segments 1–3, Abd. 1–10 – abdominal segments 1–10, setae: *as* – alar, *ps* – pleural, *eps* – epipleural, *ds* – dorsal, *lsts* – laterosternal, *eus* – eusternal, *pda* – pedal, *pds* – postdorsal, *prns* – pronotal, *prs* – prodorsal, *ss* – spiracular, *sts* – sternal, *ts* – terminal.

Body (Figs 5–9) slender, slightly curved, rounded in cross section. Prothorax slightly bigger than mesothorax; metathorax as wide as mesothorax. Abdominal segments 1–6 of almost equal length; segments 7–9 tapering gradually to the terminal parts of the body; segment 10 reduced to four anal lobes of unequal size: the biggest dorsal, the smallest ventral, both lateral equal in size. Spiracle of thorax bicameral, and of abdominal segments 1–8 annular. Chaetotaxy well developed, setae capilliform, variable in length, light yellow. Each side of prothorax with nine *prns* of unequal lengths: one long, three moderately long, five short or minute (seven of them placed on pronotal sclerite, next two close to spiracle); two *ps* (one long, one medium); and one very short *eus.* Meso- and metathorax (Fig. 5) on each side with one short *prs*, four *pds*, variable in length (*pds_1_*, *pds_3_* and *pds_4_* medium, *pds_2_* relatively short), one medium *as*, one medium and one minute *ss*, one medium *eps*, one medium *ps*, and one short *eus.* Each pedal area of thoracic segments with six *pda*, variable in length (seta “*z*” invisible). Abd. 1–7 (Figs 7–9) on each side with one short *prs*, five *pds*, variable in length (*pds_1_*, *pds_3_*, and *pds_5_* very long, *pds_2_* and *pds_4_* very short) and arranged along the posterior margin of each segment, one minute and one long *ss*, one minute and one long *eps*, one minute and one medium *ps*, one short *lsts* and two short *eus.* Abd. 8 (Figs 7–9) on each side with one short *prs*, three *pds*, variable in length (*pds_1_* and *pds_3_* very long, *pds_2_* very short) and arranged along the posterior margin of the segment, one minute *ss*, one minute and one long *eps*, one minute and one medium *ps*, one short *lsts* and two short *eus.* Abd. 9 (Figs 7–9) on each side with three *ds* (*ds_1_* and *ds_3_* very short, *ds_2_* long), all located close to the posterior margin of the segment, one short and one long *ps* and two short *sts.* Each lateral anal lobe (Abd. 10) with a pair of minute *ts*.

**Figure 10, 11.**
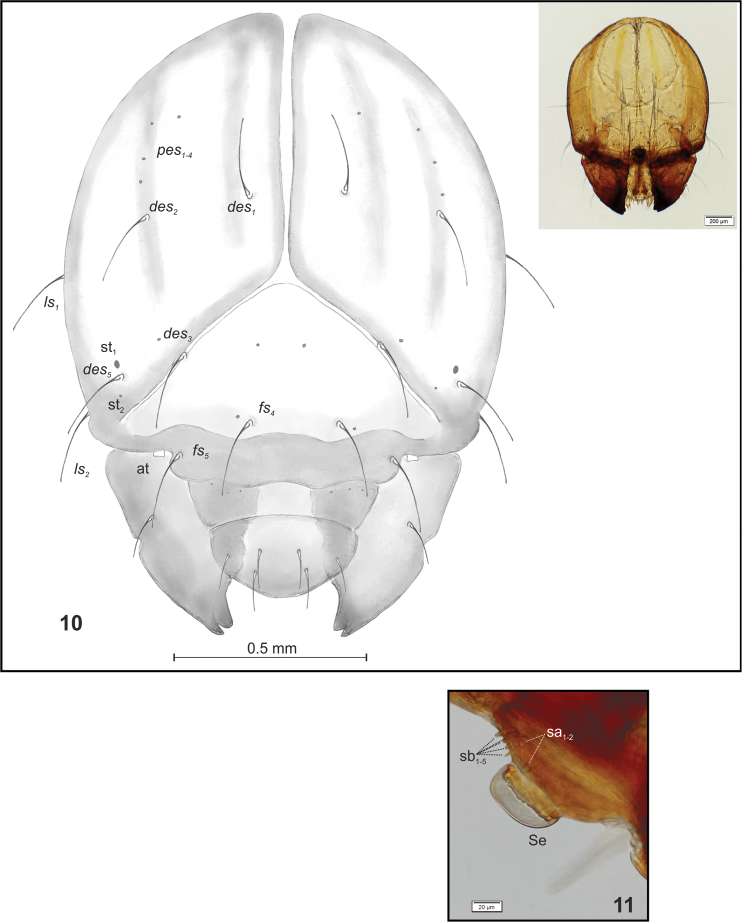
**10***Sciaphilus asperatus* mature larva, head, frontal view. Abbreviations: at – antenna, st – stemmata, setae: *des* – dorsal epicranial, *fs* – frontal, *les* – lateral epicranial, *pes* – postepicranial, *ves* – ventral **11***Sciaphilus asperatus* mature larva, right antenna. Abbreviations: se – sensorium, sa – sensillum ampullaceum, sb – sensillum basiconicum.

**Figures 12–19.**
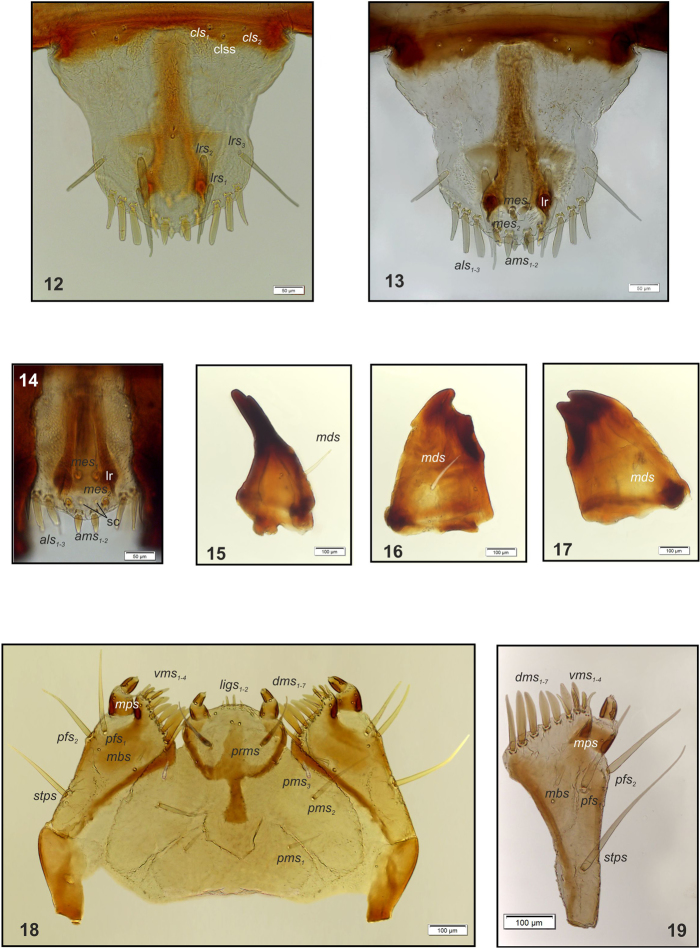
**12***Sciaphilus asperatus* mature larva, clypeus and labrum. Abbreviations: clss – clypeal sensorium, setae: *cls* – clypeal, *lrs* – labral **13***Sciaphilus asperatus* mature larva, epipharynx. Abbreviations: lr – labral rods, setae: *als* – anterolateral, *ams* – anteromedial, *mes* – median **14***Sciaphilus asperatus* mature larva, epipharynx (magnification 200×). Abbreviations: lr – labral rods, sc– sensilla cluster, setae: *als* – anterolateral, *ams* – anteromedial, *mes* – median **15–17***Sciaphilus asperatus* mature larva, right mandible **15** Lateral view **16** Dorsal view **17** Ventral view. Abbreviations: *mds* – mandibular seta **18, 19***Sciaphilus asperatus* mature larva, body parts **18** Maxillolabial complex, ventral aspect **19** Right maxilla, ventral aspect. Abbreviations: dms – dorsal malar, ligs – ligular, mbs – malar basiventral, mps – maxillary palp, pfs – palpiferal, prms – prelabial, pms – postlabial, stps – stipal, vms – ventral malar.

Head (Fig. 10) light to dark yellow with some dark, vertical stripes, suboval, slightly narrowed bilaterally; frontal suture distinct, Y-shaped, endocarina absent. Setae on head capilliform. *Des_1, 2, 3, 5_* equal in length; *des_1_* and *des_2_* located in centre of epicranium, *des_3_* placed on frontal suture, *des_4_* absent, *des_5_* located anterolaterally. *Fs_4, 5_* equal in length, *fs_4_* located anteromedially, *fs_5_* anterolaterally, close to epistoma. *Les_1_* and *les_2_* equal in length, as long as *des_1_*. Postepicranial area with four very short *pes.* A pair of small stemmata (st_1, 2_) located anterolaterally on each side of head, variable in size. Antennae (Fig. 11) inserted at end of frontal suture; antennal segment membranous with cushion–like, relatively short Se, located medially and with seven sensilla of different types: two sa and five sb. Labrum (Fig. 12) semicircular, anterior margin smooth; three pairs of *lrs* equal in length; *lrs_1_* and *lrs_2_* placed medially, *lrs_3_* laterally. Clypeus (Fig. 12) trapezoid, anterior margin of clypeus gently arcuate inwards; two pairs of *cls* strongly reduced, vestigial, located posteromedially; clss clearly visible, placed medially between *cls.* Epipharynx (Figs 13, 14) with three pairs of rod-shaped *als* of almost equal length; two pairs of *ams* rod-shaped, variable in size, both distinctly shorter than *als*; 2 pairs of rod-shaped *mes* of almost equal length: the first pair placed medially, the second pair anteriorly, very close to *ams.* There is a pair of sensilla clusters (sc) close to *mes_2_*. Labral rods elongate, curved outwards (both form a shape close to “Y”). Anterior margin of epipharynx smooth, medial part serrate due to presence of thorn-like asperities placed between labral rods. Labral rods rather short, slightly converging posteriorly. Mandibles (Figs 15–17) distinctly curved, narrow, with divided apex (teeth variable in length). A protruding additional tooth on the cutting edge between apex and middle of mandible; single *mds* capilliform, medium long. Maxilla (Figs 18, 19) with one long *stps* and two *pfs* of equal length; mala with seven finger-like *dms*, variable in size, and four *vms* (Figs 15, 16); *vms* rod-like, variable in length, always shorter than *dms*; *mbs* very short. Maxillary palpi with two palpomeres, basal with short *mps*; distal palpomere apically with a group of sensilla, each palpomere with a pore. Basal palpomere distinctly wider and longer than distal, basal to distal length ratio: 1.5:1. Prelabium (Fig. 18) almost rounded with one very long *prms*, located medially. Ligula with two pairs of capilliform *ligs* of variable length. Premental sclerite clearly visible, trident-shaped, its posterior extension truncated, expanded at apex. Labial palpi two-segmented; apex of distal palpomere with some sensilla; each palpomere with a pore. Basal palpomere wider and distinctly longer than distal, basal to distal length ratio: 2:1. Postlabium (Fig. 18) with three capilliform *pms*, the first pair located proximally, the second medially, and the third laterodistally; *pms_1_* short, *pms_2_* and *pms_3_* very long.

### Description of the pupa

All data in [mm].

Body length: 4.00^2^, 4.75^1^, 5.25^1^, 5.50^4^; body width: 3.00^3^, 3.25^2^, 3.50^3^.

Head width: 1.20^4^, 1.24^2^.

**Figures 20–22.**
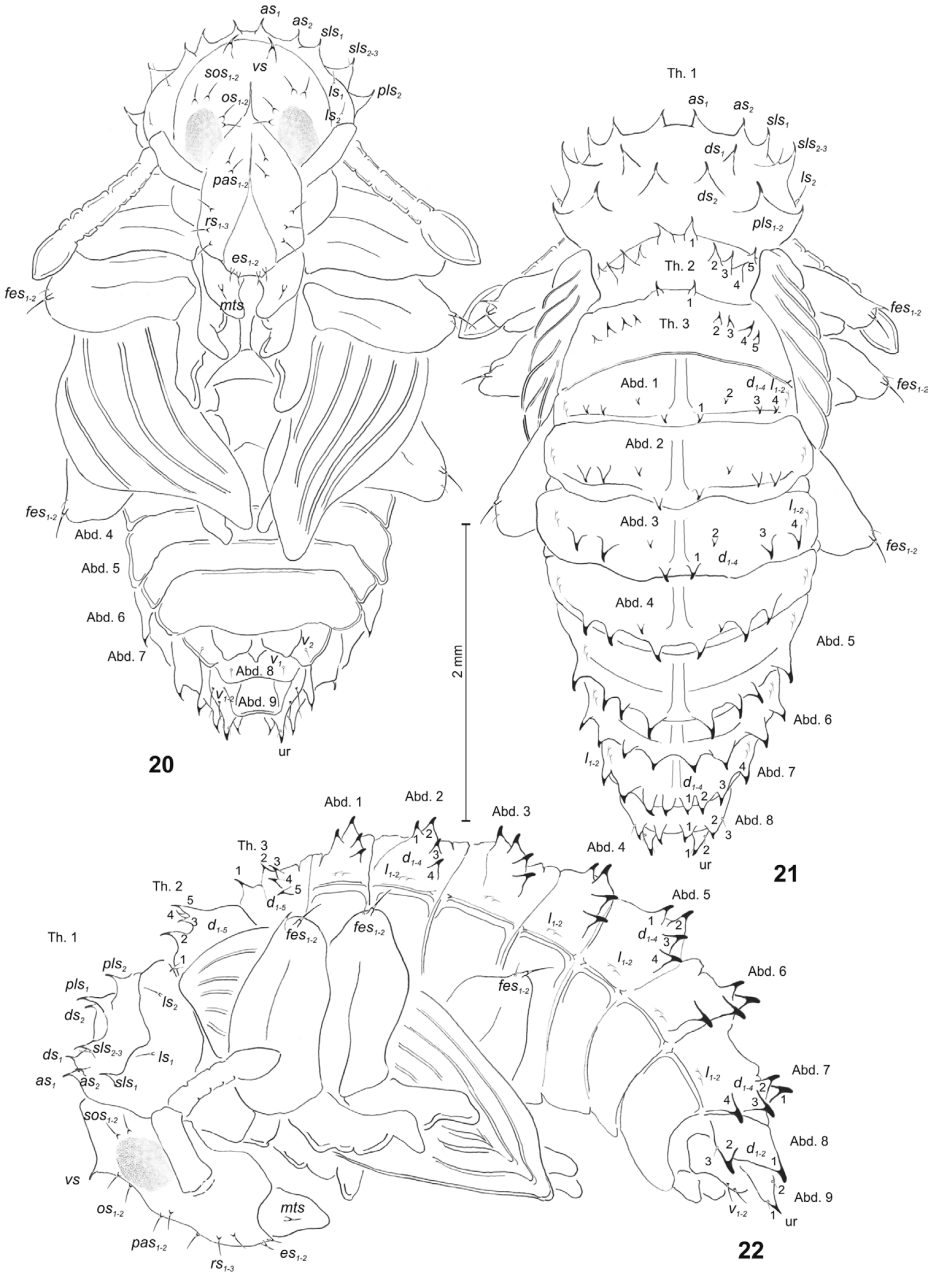
*Sciaphilus asperatus* pupa. **20** Ventral view **21** Dorsal view **22** Lateral view. Abbreviations: Th. 1–3 – pro-, meso- and metathorax, Abd. 1–9 – abdominal segments 1–9, ur – urogomphus, setae: as – apical, d – dorsal, ds – discal, es – epistomal, fes – femoral, l, ls – lateral, mts – mandibular theca, os – orbital, pas – postantennal, pls – posterolateral, rs – rostral, sls – superlateral, sos – superorbital, v – ventral, vs – vertical.

Body moderately slender, straight, whitish. Cuticle densely covered with asperites. Rostrum short, 1.3 times as long as wide, extended beyond procoxae. Antennae relatively long and slender. Pronotum almost 2.0 times as wide as long. Abdominal segments 1–3 of almost equal length, segments 4–6 tapering gradually, 7 semicircular, 8 smaller than previous segments, 9 strongly reduced. Urogomphi short, conical, slightly sclerotized at apex (Figs 20–22).

Chaetotaxy well developed, setae of various lengths and shapes: on head (except *vs*), rostrum and mandibular thecae, capilliform, straight; on dorsal parts of thoracic (except *ls*) and abdominal segments, thorn-like. Setae yellowish to brownish, usually located on visible protuberances. Head capsule and rostrum with one pair of *vs*, two pairs of *sos*, *os*, *pas*, three pairs of *rs*, and two pairs of *es. Vs* thorn-like, medium-sized; all *sos*, *os*, *pas* and *rs* medium long, straight, equal in length; *es* and *mts* straight, very short (Fig. 20). Pronotum with two pairs of *as*, *ls*, *ds*, *pls*, and three pairs of *sls.* Only *ls* and *sls_3_* thin, capilliform, remaining setae thorn-like, placed on distinct protuberances. *Sls_2_* and *sls_3_* growing together on a single protuberance (Fig. 22).

Meso- and metathorax each with five pairs of rather small setae forming a line medially. Abdominal segments 1–7 each with 4 pairs of thorn-like *ds* (placed along posterior margin), and 2 minute, capilliform *ls.* Dorsal setae on abdominal segments 1 and 2 small, equal in length, on next segments increasing gradually in size; segment 8 with two pairs of minute, capilliform *ls*, two minute, capilliform *vs*, and three pairs of *ds*: first and second thorn-like, third capilliform, *ds_2_* and *ds_3_* growing together on a single protuberance; segment 9 with two pairs of minute, capilliform *vs*, next two with minute setae on each urogomphus (Fig. 22). Apex of femora with 2 *fes*; *fes_1_* long, straight, *fes_2_* short, thorn-like, both placed on protuberances (Figs 20–22).

## Discussion

*Sciaphilus asperatus* is a common species. Biology and life cycle are in general well known. However, some special aspects of development, such as number of larval instars, voltinism and oviposition capacity have to be discussed herein. Some differences in chaetotaxy between *S. asperatus* and selected genera from Entiminae are also discussed. Finally, larva and pupa are integrated in current determination keys.

**Figures 23–26.**
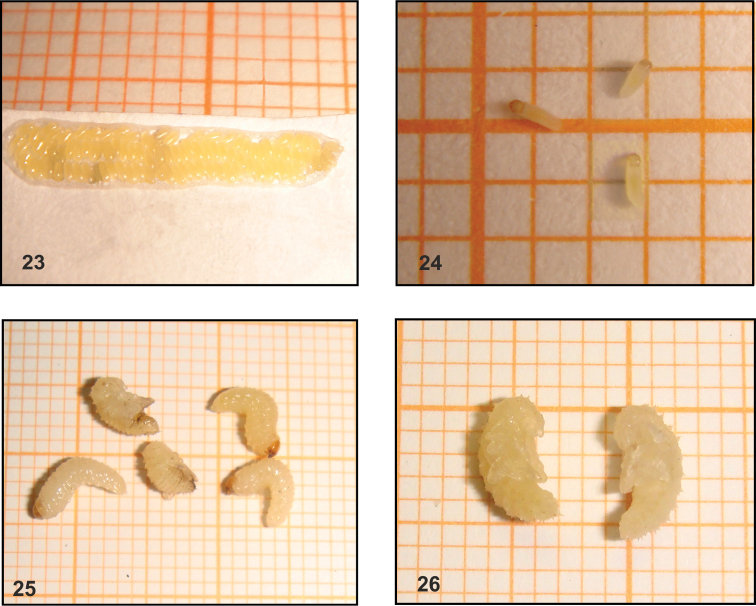
*Sciaphilus asperatus* immature stages from the laboratory (eggs, L_1_ larvae) and from the field (mature larvae, pupae). **23** Eggs **24** First instar larvae **25** Mature larvae and pupae **26** Pupae (photograph P. Sprick).

### Larval instar determination

Willis (1964) reported six larval instars, but the diagram on which the results of his measurements are based shows only five. He provides measurement data for 377 larvae. We checked this using the method of Sprick and Gosik (2014) or Gosik et al. (2019): see Tables 1, 2.

The data listed in Table 1 show that the mean values are very close for L_1_ larvae: 0.277 mm (our data) and 0.233 mm according to Emden (1952). In mature larvae the range is a little larger but also quite close: 1.117 mm (our data) and 1.215 mm (Emden 1952). The HW of six measured pupae lies within this range. These are the best pre-conditions for larval instar determination (Table 2). The tested Growth Factor (GF) values are around 1.40, as in some other species (see for example Sprick and Gosik 2014 or Gosik et al. 2019).

From Table 2 it can be inferred that larval growth is rather slow: the best approximation in both cases is achieved with GF values < 1.4: 1.37 – 1.38 (1.375) from our own data and 1.39 – 1.40 (1.391) from the data of Emden (1952). These values are much smaller than in *Tanymecus* (Gosik et al. 2019), where GF values ranged between 1.44 and 1.45. Furthermore, it is immediately obvious that *Sciaphilus asperatus* has six larval instars and that the indefinite larvae of Emden (1952) (Table 1) belong to the 4^th^ larval instar.

**Table 1.** Head width data in *Sciaphilus asperatus* immatures.

Instar	Data [mm]	Mean value (x̅) [mm]	Source
L_1_	0.22, 0.23, 0.25	0.233	Emden (1952)
L_1_	0.224, 0.23	0.227	own data
Indefinite instar	0.6, 0.67	0.635	Emden (1952)
Mature larvae	1.17, 1.26, 1.38*, 1.41*	1.215	Emden (1952)
Mature larvae	1.05, 5× 1.10, 3× 1.15, 1.17	1.117	own data
Pupa	4× 1.20, 2× 1.24	1.213	own data

*: The head widths (HW) of 1.41 and 1.38 mm are beyond the range of Willis’ data for > 280 mature larvae (Willis 1964). Hence, these two larvae are not considered typical of this species and will be excluded from the instar determination. Willis’ data for mature larvae range between ca. 0.88 mm and 1.35 mm with a maximum at 1.12 mm (71 larvae) according to his figure 45 and between 1.06 mm and 1.30 mm according to the text (page 97).

**Table 2.** Larval instar and Growth Factor determination in *Sciaphilus asperatus*.

Larval instar	GF values to be tested: 1.37/1.38/1.39/1.40/1.43	GF values to be tested: 1.37/1.38/1.39/1.40/1.43
L_1_ (measured)	**0.227** ^1)^	**0.233** ^2)^
*L_2_ (calculated)*	*0.311/0.313/0.316/0.318/0.325*	*0.319/0.322/0.324/0.326/0.333*
*L_3_ (calculated)*	*0.426/0.432/0.439/0.445/0.464*	*0.437/0.444/0.450/0.457/0.476*
*L_4_ (calculated)*	*0.584/0.597/0.610/0.623/0.664*	*0.599/0.612/0.626/0.639/0.681*
*L_5_ (calculated)*	*0.800/0.823/0.847/0.872/0.949*	*0.821/0.845/0.870/0.895/0.974*
*L_6_ (calculated)*	*1.096/1.136/1.178/1.221/1.357*	*1.124/1.166/1.209/1.253/1.393*
Mature larvae (measured)	**1.117**	**1.215**

Italics: calculated values; bold: measurements (except head line). – ^1)^: own data; ^2)^ data of Emden (1952)

### Oviposition capacity

Some data are available regarding the egg-laying capacities of *Sciaphilus asperatus*. According to Emden (1952), the volume of a female’s abdomen is 277 times that of a single egg. In actual fact, however, the available space must be less because of the space requirements of the digestive system, the ovipositor, viscose fluids, bordering structures and others. The highest recorded egg mass was 157 eggs per oviposition event (Willis 1964).

Willis (1964) and Dieckmann (1980) respectively reported ca. 880 and 1000 eggs laid by a single female during one season. But these data are from (or probably from) weevils maintained in the laboratory. The data relating to weevils maintained under outdoor conditions by Willis (1964) resulted in lower values of 450 to 700 eggs per female. If the pre-oviposition period lasts around 10 days (see Willis 1964), there could be 12 egg deposition events between mid-April and the end of July. If this is right, 38 to 60 eggs could be laid per oviposition event under outdoor conditions.

### Generations and voltinism

According to the data presented by Krause (1978) and Dieckmann (1980), *Sciaphilus asperatus* should be a univoltine species: overwintered adults produce eggs, larvae hatch in spring and summer, pupation takes place in summer, and adult weevils of the new generation emerge also in summer. But Burakowski et al. (1993) reported that a small part of the new generation lays eggs in August, producing larvae that overwinter. Moreover Willis (1964) presumed that usually all larvae overwinter. This hypothesis has still to be checked. It appears equally possible that larvae from eggs laid early in the season develop in the same year as is true for many other soil-dwelling weevils (see for example Gosik et al. 2016, Gosik and Sprick 2017).

A species that develops within one season is univoltine, whereas a species that needs longer than one year for its development is semivoltine. Neither definition fits *S. asperatus* or many other soil-dwelling weevils. Apparently, there is a mix of univoltine summer development, and univoltine or semivoltine (if development of the overwintering larvae should last longer than one year) autumn/spring development; in winter there is not usually any development. It seems these definitions are hard to apply to soil-dwelling weevils, as they do not fit the facts very well.

### Remarks on chaetotaxy

There are only several small discrepancies between the description of the mature larva given by Emden (1952) and those presently described: e.g. Emden reported two mandibular setae (one prominent and next very small), whereas we observed only a single seta. It is possible that the second (very small) *mds* visible on the first instar larva was torn off during intensive feeding of the mature larva. Emden (1952) reported on the presence of seven setae on the pedal area of the prothorax, the seventh a minute seta (“*z*”), and three further minute setae of each lateral anal lobe. We noticed only six setae on each pedal lobe and only two pairs of minute terminal setae on the lateral lobes of the tenth segment.

It is worth stressing that the presently described mature larva of *S. asperatus* possesses exactly all essential characters listed by Marvaldi (1998) for Entiminae larvae, Type “A”, namely: single *as*; setae *mes_1_* close together whereas setae *mes_2_* placed far one another; *mes_2_* placed close to *ams*; sensilla cluster placed between *mes_2_*; labral rods curved outwards; premental sclerite trident-shaped, with posterior extension truncate, expanded at apex.

### Supplement to the key to selected genera and tribes of Palaearctic Entiminae larvae

Based on Gosik et al. (2016), Gosik and Sprick (2017), Gosik et al. (2017), and Gosik et al. (2019): in *Graptus, Peritelus*, and *Sciaphilus* the key is based on one species each (*G. triguttatus triguttatus*, *P. sphaeroides*, and *S. asperatus*).

(Previous step as in Gosik et al. 2019)

**Table:** 

2	Abdominal segment 10 reduced to three lobes; clypeus always with well-developed median furrow; meso- and metathorax each with single *ss (sps)*	**Sitonini (*Andrion*, *Charagmus*, *Coelositona*, *Sitona*)**
(next steps as in Gosik and Sprick 2017)		
–	Abdominal segment 10 reduced to four lobes; clypeus smooth; meso- and metathorax each with 2–3 *ss* (*sps*)	**2a**
2a	All spiracles (thoracic and abdominal) bicameral	***Otiorhynchus* (Otiorhynchini)**
(next steps as in Gosik et al. 2016)		
–	At least abdominal spiracles annular	**2b**
2b	Meso- and metathorax with 3 *ss* each, Se conical	***Graptus* (Byrsopagini)**
–	Meso- and metathorax with 2 *ss* each, Se cushion-like	**2c**
2c	Head unicolour. All spiracles annular. Each pedal area with 4 *pda*, abdominal segment 8 with 4 *prs*; abdominal segment 9 with 4 *ds*	***Peritelus* (Peritelini)**
–	Head with faint stripes. Thoracic spiracles bicameral, abdominal annular. Each pedal area with 6 *pda*, abdominal segment 8 with 3 *prs*; abdominal segment 9 with 3 *ds*	***Sciaphilus* (Sciaphilini)**
3	All spiracles (thoracic and abdominal) bicameral; head oval	***Strophosoma* (Brachyderini)**
(next steps as in Gosik et al. 2017)		
–	All spiracles (thoracic and abdominal) annular; head narrowed bilaterally	***Philopedon* (Cneorhinini), *Tanymecus* (Tanymecini)**
(next steps as in Gosik et al. 2019)		

### Upgrade to the key to pupae of selected Palaearctic Entiminae genera and tribes by Gosik and Sprick (2013)

(Previous steps as in the original key)

**Table:** 

9	Pronotal and abdominal setae thorn-like, inserted on elongate protuberances	**Sciaphilini Sharp, 1891**
9a	Body length in both sexes < 4.75 mm, head with 1 pair of *os*, rostrum with 4 pairs of *rs*, mandibular thecae without setae; *as_1_* distinctly smaller than *as_2_*, pronotum with 1 pair of *ls* and 2 pairs of *sls*; each abdominal segment 1–7 with 3 pairs of setae, *fes_1_* and *fes_2_* equal in length	***Exomias* Bedel, 1883 (= *Barypeithes* Du Val, 1854, in part)**
9b	Body length of females up to 5.50 mm (usually > 4.75 mm), head with 2 pairs of *os*, rostrum with 3 pairs of *rs*, mandibular thecae with 1 pair of *mts*, *as_1_* and *as_2_* equal in size, pronotum with 2 pairs of *ls* and 3 pairs of *sls*, each abdominal segment 1–7 with 4 pairs of setae, *fes_1_* distinctly bigger than *fes_2_*	***Sciaphilus* Schönherr, 1823**

Taking into consideration the shape, number, and distribution of setae, and the general body shape, the pupae of *Sciaphilus asperatus* and of *Exomias pellucidus* (Boheman, 1834) are very similar (see Gosik and Sprick 2013). Especially due to hair-like setae on head and rostrum and to thorn-like setae on pronotum and abdomen, which are observed on both species as well as the presence of paired *sls* growing on single protuberances and the absence of ventral setae on abdominal segments 1–7. This morphological information is coherent with the close systematic position of both species in the tribe Sciaphilini.
